# Chitosan modified Fe_3_O_4_/KGN self-assembled nanoprobes for osteochondral MR diagnose and regeneration

**DOI:** 10.7150/thno.43569

**Published:** 2020-04-15

**Authors:** Yuping Hong, Yaguang Han, Jun Wu, Xinxin Zhao, Jin Cheng, Guo Gao, Qirong Qian, Xiuying Wang, Weidong Cai, Hala Zreiqat, Dagan Feng, Jianrong Xu, Daxiang Cui

**Affiliations:** 1 Institute of Nano Biomedicine and Engineering, Shanghai Engineering Research Centre for Intelligent Diagnosis and Treatment Instrument, Department of Instrument Science and Engineering, School of Electronic Information and Electrical Engineering, Shanghai Jiao Tong University, 800 Dongchuan RD, Shanghai 200240, PR China; 2 Institute of Nano Biomedicine, National Engineering Center for Nanotechnology, 28 Jianchuan East RD, Shanghai 200241, PR China; 3 Department of Joint Surgery and Sports Medicine, Changzheng Hospital, Second Military Medical University, 415 Fengyang RD, Shanghai 200003, PR China; 4 Department of Radiology, Ren Ji Hospital, School of Medicine, Shanghai Jiao Tong University, 160 Pujian RD, Shanghai 200127, PR China; 5 School of Computer Science, Faculty of Engineering, University of Sydney, NSW 2006, Australia; 6 Murray Maxwell Biomechanics Laboratory, Kolling Institute, Royal North Shore Hospital, University of Sydney, NSW 2065, Australia

**Keywords:** Fe_3_O_4_-CS/KGN nanoprobe, self-assembly, theranostic strategy, MRI, osteochondral regeneration therapy

## Abstract

Chondral and osteochondral defects caused by trauma or pathological changes, commonly progress into total joint degradation, even resulting in disability. The cartilage restoration is a great challenge because of its avascularity and limited proliferative ability. Additionally, precise diagnosis using non-invasive detection techniques is challenging, which increases problems associated with chondral disease treatment.

**Methods:** To achieve a theranostic goal, we used an integrated strategy that relies on exploiting a multifunctional nanoprobe based on chitosan-modified Fe3O4 nanoparticles, which spontaneously self-assemble with the oppositely charged small molecule growth factor, kartogenin (KGN). This nanoprobe was used to obtain distinctively brighter T_2_-weighted magnetic resonance (MR) imaging, allowing its use as a positive contrast agent, and could be applied to obtain accurate diagnosis and osteochondral regeneration therapy.

**Results:** This nanoprobe was first investigated using adipose tissue-derived stem cells (ADSCs), and was found to be a novel positive contrast agent that also plays a significant role in stimulating ADSCs differentiation into chondrocytes. This self-assembled probe was not only biocompatible both *in vitro* and *in vivo*, contributing to cellular internalization, but was also used to successfully make distinction of normal/damaged tissue in T_2_-weighted MR imaging. This novel combination was systematically shown to be biosafe via the decrement of apparent MR signals and elimination of ferroferric oxide over a 12-week regeneration period.

**Conclusion:** Here, we established a novel method for osteochondral disease diagnosis and reconstruction. Using the Fe_3_O_4_-CS/KGN nanoprobe, it is easy to distinguish the defect position, and it could act as a tool for dynamic observation as well as a stem cell-based therapy for directionally chondral differentiation.

## Introduction

Chondral damage due to trauma or pathologic changes commonly leads to cartilage degeneration and osteoarthritis development, eventually resulting in total joint degradation. The restoration of articular cartilage defects is clinically challenging because its highly organized layered structure and avascularity hamper recovery 1, 2. It is suggested that appropriate intervention in the early stages could postpone progressive destruction in articular cartilage diseases. However, generally, joint cartilage deterioration is difficult to effectively verify and treat as it requires precise imaging technologies and noninvasive inspection 3, 4. In the past several decades, magnetic resonance imaging (MRI) has been widely employed in clinical joint injury diagnosis and plays an indispensable role in noninvasive chondral damage detection through multidirectional scanning and 3D reconstruction. Even though significant improvements have been developed, such as higher field intensity, sensitive coils and advanced pulse sequences, it is still not possible to properly distinguish the subtle cartilage structure 3 and the artifacts caused by partial volume effects during the precise cartilage defect inspection 5, 6. Paramagnetic or superparamagnetic nanoparticles (MNPs), such as gadopentetate dimeglumine (Ga-DTPA) 7, copper sulphide (CuS) 8 and ferroferric oxide (Fe_3_O_4_) 9, could be used as MR contrast agents. Some of them can even integrate multi-mode imaging technologies, and are reliably used in deep-tissue imaging 10.

Superparamagnetic Fe_3_O_4_ MNPs have been intensively developed for use in some areas, such as magnetic sensors 11, storage media 12, medical applications 13 and anti-infection nanozyme 14. Notably, Fe_3_O_4_ is regarded as a biocompatible drug carrier, and has been approved by the FDA in both clinical research and treatment. Currently, modified Fe_3_O_4_ MNPs are used as drug vehicles 13, MRI agents 15, and tracers 16* in vitro* or* in vivo*, where they usually influence transverse relaxation and act as negative contrast agents 17. Even though Fe_3_O_4_ offers various benefits, it is still sensitive to magnetization and oxidation 18, therefore a superficial coating is essential for protection and stability.

To improve tissue regeneration, composite materials have been used in tissue engineering, such as strontium-graphene oxide (Sr-GO) nanocomposites 19. However, they are usually resistant to breakdown in the human body. Chitosan is a natural polysaccharide and has attracted extensive interest because of its reasonable cost, biodegradability and biocompatibility. Conjugating a macromolecular skeleton onto MNPs contributes to electronic stability as well as pharmacodynamics and relaxivity 20, 21. In addition, the deacetylated products of chitosan are rich in exposed amino groups, providing potential reactive sites and preventing metal sedimentation by charge repulsion. Preparation made by chemically coupling chitosan tends to improve solubility, accelerates cellular penetration and internalization, and even changes the targeting sites 22. Thus, chitosan is a promising macromolecule for coating the core of metal oxides.

As an aromatic and drug-like compound, kartogenin (KGN) was initially screened out as a growth factor contributing to chondrogenesis and chondroprotection that works in a dose-dependent manner 23. In the presence of KGN, filamin A is known to disrupt the interaction with the core-binding factor β subunit (CBFβ), leading to stimulation of mesenchymal stem cells (MSCs) and chondrogenesis 2, 23, 24. Unlike the readily degradable bio-macromolecular growth factors, KGN is reasonably chemically stable in solution. In addition, this cytokine cocktail enables a reduction of nitric oxide (NO) and free glycosaminoglycans (GAGs) on inflamed and damaged chondrocytes 23, 24.

Recently, great achievements have been realized in the area of magnetic iron oxide nanoparticles for noninvasive *in vivo* diagnosis. In the clinic, Gd and Fe are usually employed as basic elements for contrast agents in different MRI scanning patterns. Gd chelators are used as positive T_1_-weighted contrast agents due to a decrease in the spin-lattice relaxation time 25. Mn is another element for MR imaging, which can be used as a T_1_-weighted contrast agent 26. However, some drawbacks still need to be addressed, such as renal toxicity and tissue accumulation 17, 27. Fe, as an intrinsic element of the human body, has been approved by the FDA for clinical application. Differing from Gd chelates, Fe contrast agents display superparamagnetism and usually provide dark T_2_-weighted imaging. In some case, Fe^2+^ ions from Fe agents gradually transform into Fe^3+^, resulting in positive and brighter images 28.

Based on the above rationale, we designed a novel magnetic Fe_3_O_4_ nanoprobe for joint cartilage with distinctively enhanced brighter T_2_-weighted contrast effects. In this study, oleic acid-modified ferroferric oxide (Fe_3_O_4_ oleic acid) was further grafted with positive-charge bearing chitosan (Fe_3_O_4_-CS), which then self-aggregated by the interplay between it and negatively charged KGN, and by hydrophobic interactions, causing its assembly into larger superparamagnetic nanoparticles (Fe_3_O_4_-CS/KGN). These superparamagnetic nanoparticles exhibited brighter T_2_-weighted enhancement *in vitro* and *in vivo*, due to an enclosed Fe_3_O_4_ core and KGN causing ferric electric charge transfer. Furthermore, Fe_3_O_4_-CS/KGN was also verified as an effective medical carrier that stimulated adipose tissue-derived stem cells (ADSCs) to increase type 2 collagen (Col Ⅱ) secretion and chondrogenic differentiation. This carrier was proven susceptible to elimination *in vivo*, since the enhanced bright ferroferric oxide signals were hardly detectable after 12 weeks. Additionally, Fe_3_O_4_-CS/KGN also played a protective and restorative role in cartilage regeneration, leading to lesion restoration in osteochondral lesions. This study describes the mechanism of Fe_3_O_4_ self-assembly formation as well as a distinctly brighter T_2_-weighted imaging, which strongly suggests the potential application of the magnetic probes in diagnosis and regeneration of cartilage.

## Results and Discussion

### Characterization of Fe_3_O_4_-CS/KGN nanoparticles

**Figure 1:**
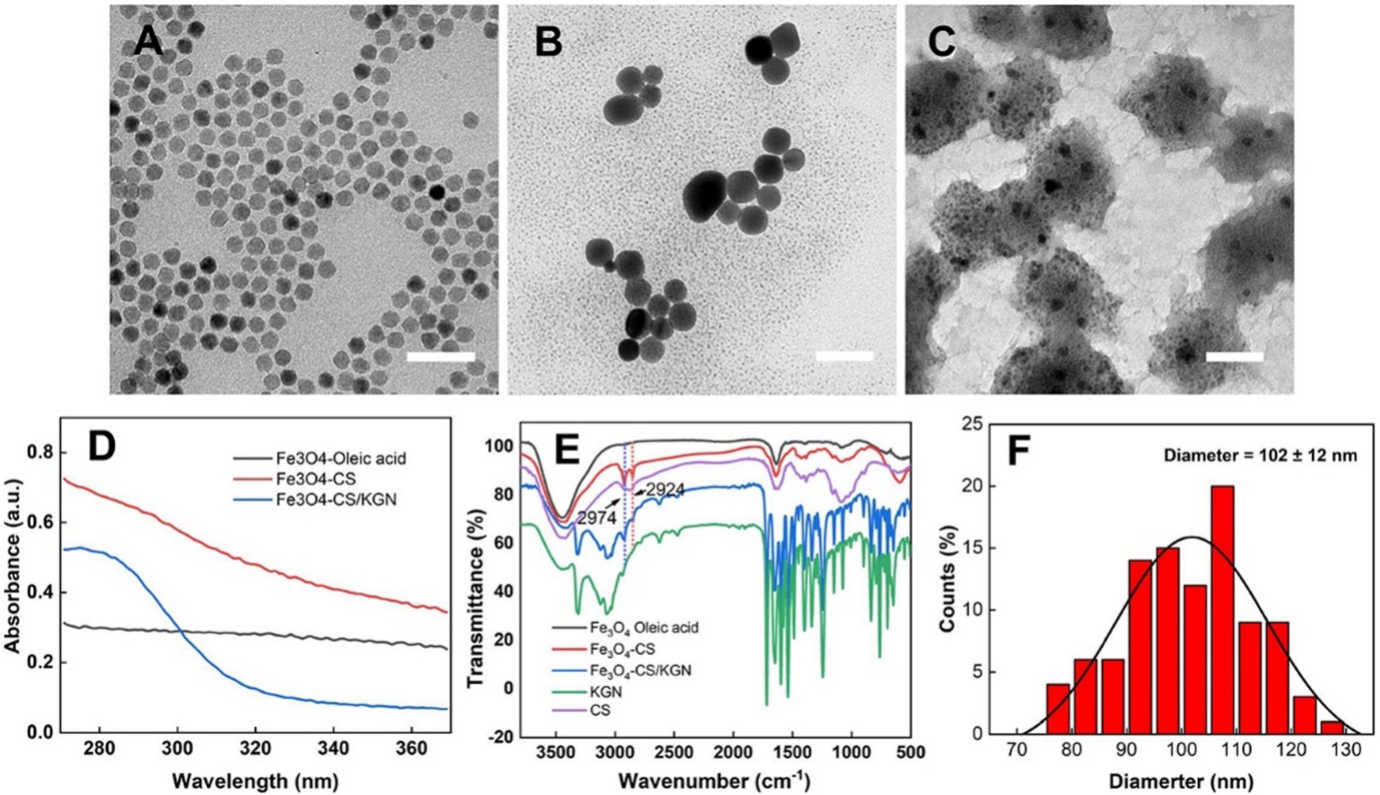
** Characterization of Fe_3_O_4_-CS/KGN. A)**, **B)** and **C)** corresponde to the mophology of the Fe_3_O_4_ oleic acid, the Fe_3_O_4_-CS and the Fe_3_O_4_-CS /KGN nanoparticles, respectively. Scale bar is 50 µm; **D)** The UV-Vis spectra; **E)** the FT-IR spectrum; **F)** the diameter distribution of the Fe_3_O_4_-CS /KGN nanoparticles (n = 100).

The Fe_3_O_4_-CS/KGN MNPs were synthesized via modification of a condensation reaction and self-assembly. As shown in Figure 1, the original Fe_3_O_4_ oleic acid MNPs were successfully grafted with chitosan via EDC/sulfo-HOSu, leading to an increase in diameter from 10 nm to 10-20 nm (Figure 1A and 1B). As shown by transmission electron microscopy (TEM, Tecnai G2 Spirit BioTwin, USA) images, Fe_3_O_4_ was encapsulated with a thick CS shell, which prevented the Fe_3_O_4_ from aggregating. In order to load KGN into Fe_3_O_4_ MNPs, the monomer Fe_3_O_4_-CS particles were aggregated to form larger assembled Fe_3_O_4_-CS/KGN MNPs (Figure S1, confirmed by nuclear magnetic resonance, NMR, Avance Ⅲ, 600 MHz, Bruker, Germany), which had a general diameter of 102 ± 12 nm 29 (Figure 1C and F). In order to explore the spectroscopic characteristics of the material, UV-Vis and Fourier transform infrared (FT-IR) spectroscopy were employed. As shown in Figure 1D, Fe_3_O_4_-oleic acid and Fe_3_O_4_-CS had no apparent absorbance peaks, while the self-assembled Fe_3_O_4_-CS/KGN had a strong, broad absorbance peak at approximately 283 nm, indicating that KGN was attached to the Fe_3_O_4_-CS nanoparticles and induced Fe_3_O_4_-CS to grow into larger Fe_3_O_4_-CS/KGN nanoparticles. In the FT-IR spectrum (Figure 1E), the peaks between 2924 cm^-1^ and 2973 cm^-1^ were present in Fe_3_O_4_-CS as well as in Fe_3_O_4_-CS/KGN, while CS showed weak, broad peaks in the same positions. Additionally, several strong peaks in the region 1700-500 cm^-1^ were observed, indicating that KGN was loaded into Fe_3_O_4_-CS/KGN MNPs. The loading amount was calculated to be 32% according to the standard KGN curve.

In addition, the behavior of KGN release was also explored (Figure S2). In the first 6 hours, KGN was slightly released from Fe_3_O_4_-CS/KGN at both pH5.5 and pH7.4. Over the next 2 days, the free KGN gradually increased, and the cumulative amount of released KGN at pH 5.5 was more than that at pH 7.4.

The possible reasons for KGN-induced self-assembly are as follows: Firstly, CS is a positively charged polymer that promotes tissue penetration 30. Conversely, KGN is an organic molecule that bears negative charges due to the carboxyl residue. In this way, the amino group interacts with the carboxyl residue on KGN, causing their intimate integration, this could result in hydrogen bond formation 31. Another factor may be the structural rigidity of KGN, which is inclined to hydrophobically interact with the alkane chains of CS. Therefore, monodispersed Fe_3_O_4_-CS nanoparticles aggregate via combination with KGN molecules.

### The T_2_-weighted contrast agent of Fe_3_O_4_-CS/KGN *in vitro*

**Figure 2:**
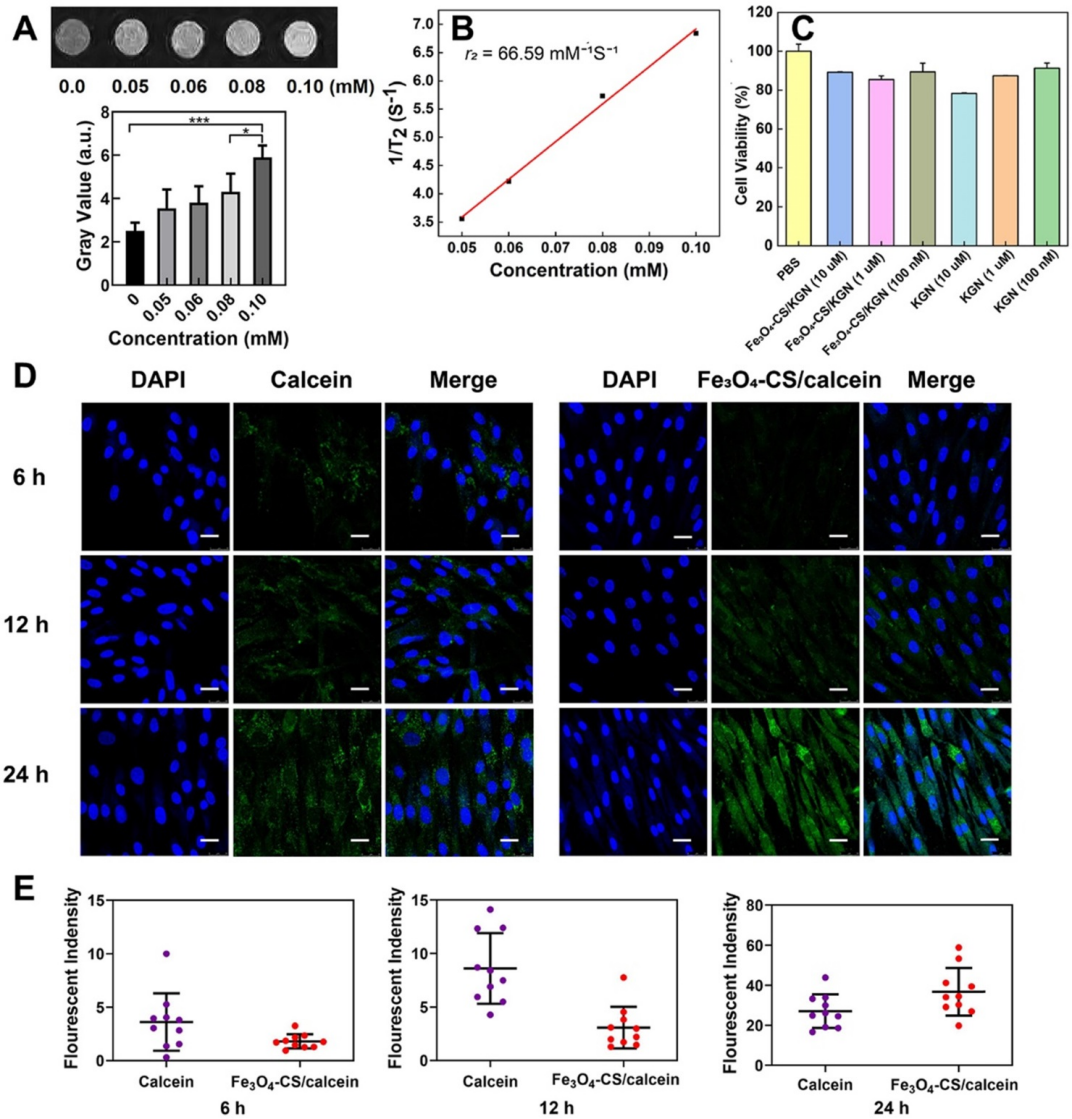
** Magnetic and cellular characterization of Fe_3_O_4_-CS/KGN. A)** T_2_-weighted MR images of different Fe_3_O_4_-CS /KGN concentrations *in vitro* (Fe concentrations), * *P* < 0.05, ** *P* < 0.01, ***P < 0.001, ns: not significant; **B)** 1/T_2_ against Fe concentrations; **C)** the CCK-8 cell toxicity assays in the presence of different Fe_3_O_4_-CS /KGN or KGN concentrations; **D)** confocal images of ADSCs exposed to 20 μg/mL Fe_3_O_4_-CS/calcein or free calcein at 6 h, 12 h and 24 h (all scale bars are 25 μm);** E)** fluorescence quantification of the internalization by ADSCs after 6 h, 12 h and 24 h incubation (n = 10).

As shown in Figure 2A, with increasing Fe concentrations, the T_2_-weighted MRI contrast effect was significantly enhanced in low field NMR (LF-NMR, MesoMR23-060H-I, China). Of note, the signal became brighter with increasing concentrations. For further quantification, the gray value of each concentration was calculated. It was found that the MR signals became brighter with the increasing Fe concentrations. In order to investigate the T_2_ relaxation, several concentrations of Fe, from Fe_3_O_4_-CS/KGN, were employed to fit the curve (1/T_2_ against Fe concentrations), and the *r_2_* value was calculated as 66.59 mM^-1^s^-1^ according to the slope of the corresponding fitting line (Figure 2B). At the molecular level of magnetic resonance, the T_2_ contrast enhancement principle of superparamagnetic Fe_3_O_4_ is explained by the outer sphere model. In general, T_2_ relaxation is dominated by native superparamagnetism, and it is also related to the protonic effective diffusion in the outer sphere 17, 32. For Fe_3_O_4_-CS/ KGN nanoparticles, CS was grafted on the surface of Fe_3_O_4_-oleic acid and there was a thick polymer shell formation, which encapsulated the metal core and limited random water movement. Thus, the protons from H_2_O were expelled from the Fe_3_O_4_ cores. Furthermore, the charges of carboxyl groups on KGN easily occupied empty orbitals from the Fe_3_O_4_. Additionally, the aryl skeleton of KGN enabled the inner water proton to remain out of the sphere core, which in turn intensified the hydrophobicity and enhanced the T_2_-weighted imaging.

### The biocompatibility and cellular uptake of Fe_3_O_4_-CS/KGN *in vitro*

To validate the biocompatibility of Fe_3_O_4_-CS/ KGN nanoparticles *in vitro*, CCK-8 assays were applied to investigate the cell viability. Firstly, as shown in Figure S3, we extracted the ADSCs and examined the differentiation antigens of ADSC characterization, such as CD90, CD44 and CD11b. The CCK-8 assay results revealed that neither Fe_3_O_4_-CS/ KGN nor KGN exhibited a significant cellular toxicity in ADSCs. As shown in Figure 2C, even high concentrations of Fe_3_O_4_-CS/KGN MNPs at 10 μM had a negligible inhibitory effect at 24 h. Incubation of Fe_3_O_4_-CS/KGN at different concentrations of 10 μM, 1 μM and 100 nM with ADSCs were all biocompatible. In addition, an Annexin/PI apoptosis kit was employed by flow cytometry (FCM, BD FACS Calibur system, USA), and the result also showed the negligible cytotoxicity *in vitro*. As shown in Figure S4, few apoptotic cells were found in corresponding quadrants. The calcein-AM/PI staining (Figure S5, observed by laser scanning confocal microscope, LSCM, TCS SP8, Leica, Germany) also indicated that few PI marked red cells were captured, suggesting high cellular viability. All the experimental results indicated good biocompatibility of Fe_3_O_4_-CS/KGN MNPs *in vitro*.

In order to verify the higher efficiency of cellular uptake, 6, 12 and 24 h LSCM observations were conducted with 20 μg/mL calcein or Fe_3_O_4_-CS/ calcein 33. In Figure 2D and 2E, after 6 h incubation, calcein treatment resulted in a higher fluorescence signal, but only slight intracellular fluorescence was detected when cells were incubated with Fe_3_O_4_-CS/ calcein. The relative fluorescent quantification ratio was approximately 4:2; at 12 h, the fluorescence intensity of the Fe_3_O_4_-CS/calcein group increased, while it was still weaker than that of calcein, and the ratio of fluorescence intensity further increased to 8:3. However, at 24 h, the ratio of fluorescence intensity of calcein and Fe_3_O_4_-CS/calcein reversed to 24:38. Eventually, free calcein was released from Fe_3_O_4_-CS/ calcein, and the fluorescence intensity was greater compared with the other groups, suggesting that the self-assembly increased cellular internalization. Once internalized in the lysosome, the free drug was gradually liberated from the assembled nanoparticles at a low pH 33.

### The intercellular distribution of Fe_3_O_4_-CS/KGN MNPs and stimulating differentiation potential

**Figure 3:**
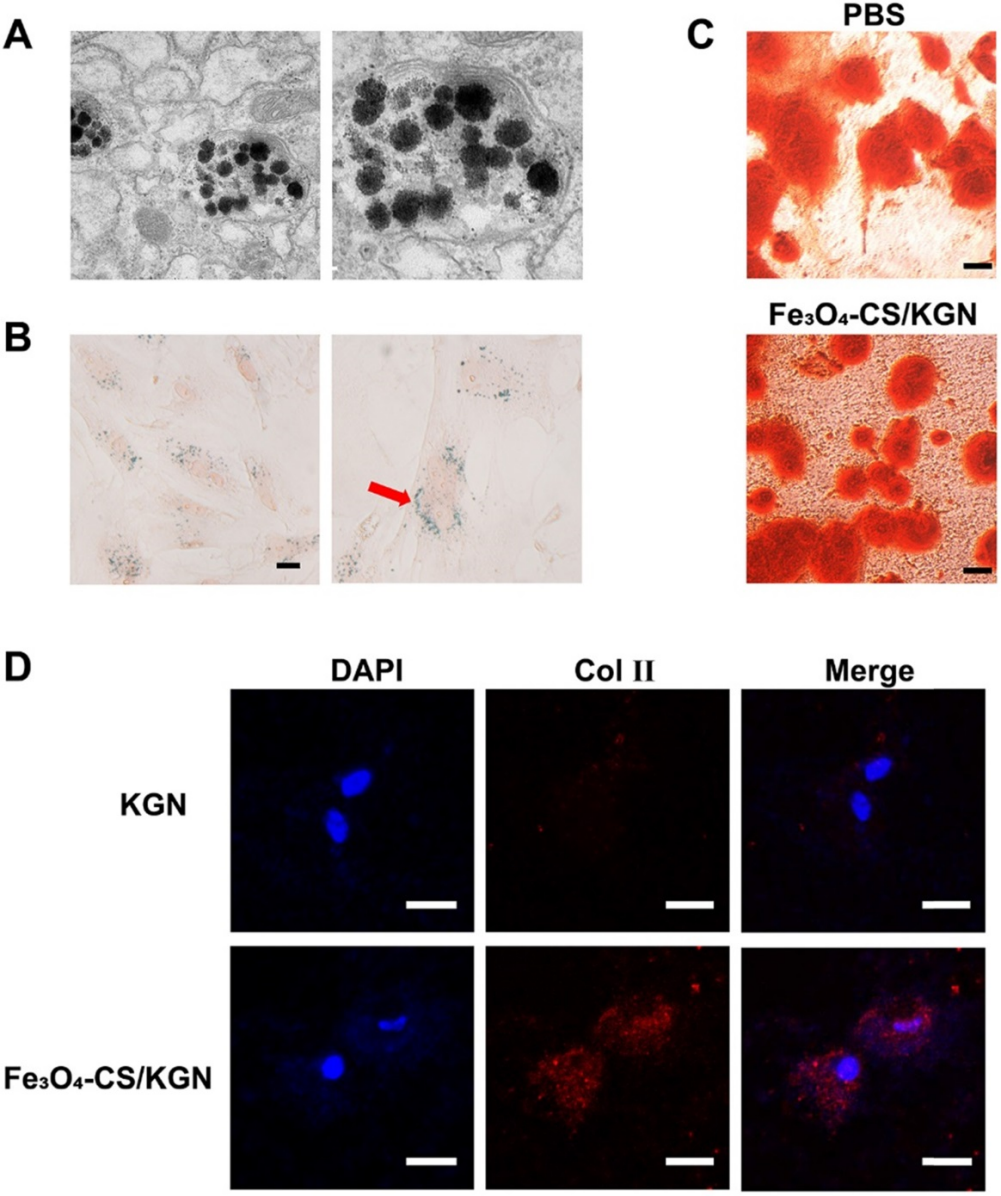
** Intracellular distribution and stimulating differentiations of ADSCs. A)** the TEM images of Fe_3_O_4_-CS /KGN inside the lysosomes of ADSC (24 h); **B)** Prussian Blue staining images for the distribution of Fe_3_O_4_-CS /KGN after 24 h (scale bars is 20 µm); **C)** the osteogenic stimulating differentiation with PBS or Fe_3_O_4_-CS /KGN *in vitro* (4 weeks, Alizarin Red S staining, scale bar is 20 µm); **D)** the type 2 collagen immunofluorescent images of induced ADSCs by 10 µM KGN or Fe_3_O_4_-CS /KGN *in vitro* (2 weeks). All scale bars are 20 µm.

As depicted in Figure 3A, the intracellular distribution of Fe_3_O_4_-CS/KGN MNPs was visualized via TEM. After 24 h incubation, partly self-assembled MNPs and dissociated Fe_3_O_4_ particles were co-encapsulated in lysosomes, indicating that the magnetic beads were easily internalized via the clathrin-lysosome route 34 and subsequently degraded. Prussian Blue staining is a typical ferric dye, and [Fe(CN)_6_]^4-^ compound from Prussian Blue combines with Fe, leading to the formation of deposit. As shown in Figure 3B, Prussian Blue deposits were extensively distributed in the cytoplasm, while the cytomorphology remained unchanged.

In this study, we introduced Fe_3_O_4_-CS/KGN to mediate ADSC chondrogenesis. In a 2-week stimulating differentiation experiment, ADSCs generated type 2 collagen (Col Ⅱ) as evidenced by their immunofluorescence. We found that a greater expression level of Col Ⅱ was detected after the treatment with 10 μM Fe_3_O_4_-CS/KGN than that of the KGN group (Figure 3D). This result suggested that Fe_3_O_4_-CS/KGN can promote ADSCs to develop into chondrocyte-like phenotypes.

Cartilage injury is always accompanied by a damaged calcified area 35. According to the knee osteochondral defect model in the calcified zone, even subchondral bone is impaired. The functions of subchondral bone are involved mechanical stress and support 36. In some cases, subchondral bone deficiency may lead to superficial cartilage layer regeneration failure 37. Sometimes, in different metabolic environments, osteogenesis and chondrogenesis differentiation compete with each other 38. Here, we also compared the potential of affecting osteogenesis in the presence/absence of Fe_3_O_4_-CS/ KGN MNPs in an osteoblast-inducing conditioning medium over 4 weeks. As shown in Figure 3C, the equivalent mineralized sediments (orange sediments, Alizarin red S staining) were deposited in the extracellular matrix (ECM) both in PBS alone and Fe_3_O_4_-CS/KGN groups. Therefore, this system has an insignificant impact on the mineralization process and osteogenesis.

The potentials of including chondrogenesis and osteogenesis indicated that the Fe_3_O_4_-CS/KGN can be used to efficiently promote either hyaline cartilage formation or osteogenic maintenance. On one hand, KGN as the chondrogenic molecule, which relies on a more effective drug-delivery path, fosters chondral formation by regulating the CBFb-RUNX1 pathway to a larger extent 2, 23, 24. On the other hand, this drug delivery system does not inhibit the osteogenic activity of ADSCs. KGN was reported to potentially accelerate osteogenesis via the regulation of silent information regulator type 1 (SIRT1) 39. As a consequence, KGN-loaded Fe_3_O_4_ MNPs enhanced intercellular antioxidant effects and maintained osteogenic capacity. However, the mechanism of Fe_3_O_4_-CS/KGN accelerating chondrogenesis but not affecting osteogenesis remains to be clarified.

### T_2_-weighted imaging using Fe_3_O_4_-CS/KGN MNPs *in vivo*

**Figure 4:**
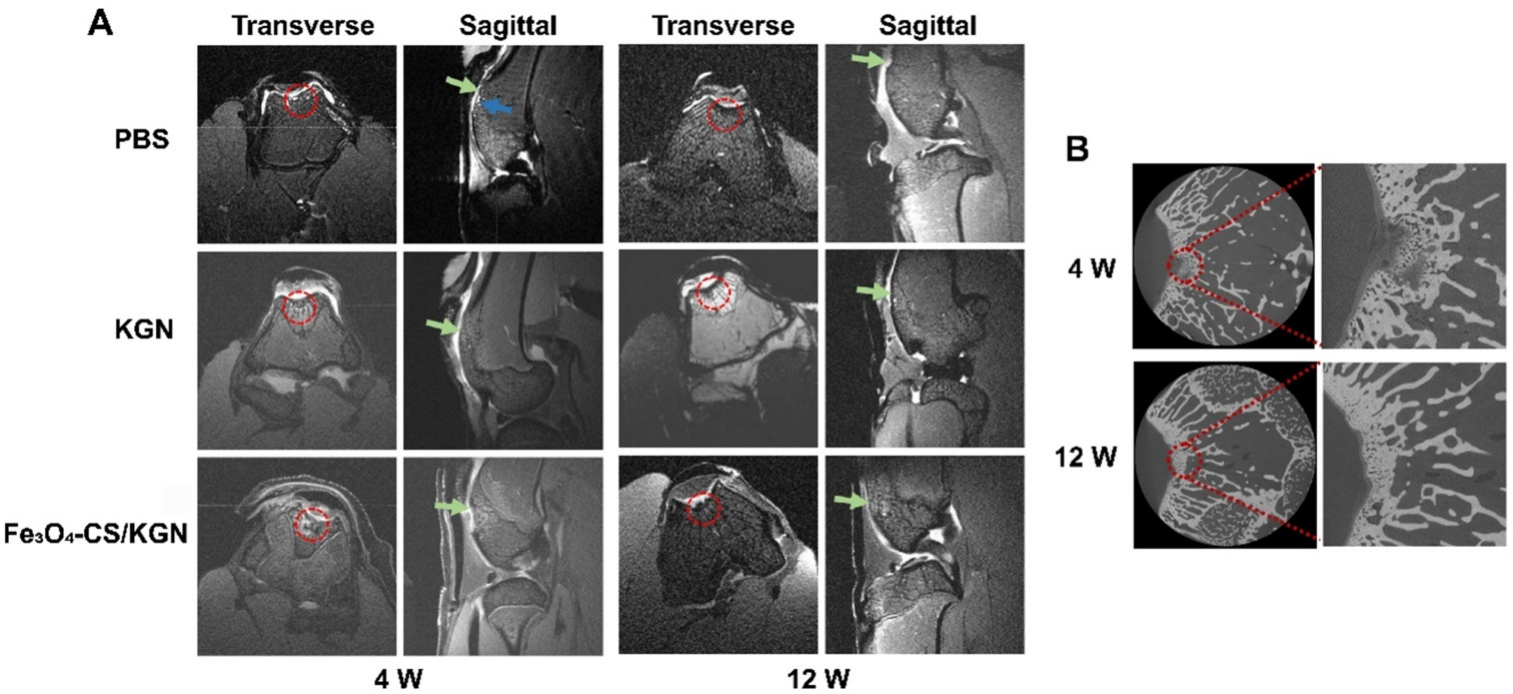
** MRI and micro-CT diagnose *in vivo*. A)** the T_2_-weighted MR images (red circle: defect site; blue arrow: edema signals; green arrow: newly formed cartilage); **B)** micro-CT images of rabbit knees after treatment with Fe_3_O_4_-CS /KGN (W = weeks).

Although the biocompatibility and biosafety Fe_3_O_4_-CS/KGN MNPs were demonstrated in T_2_-weighted imaging *in vitro*, the effectiveness of the MNPs as both contrast and therapeutic agents was further investigated *in vivo*. Firstly, a rabbit model was established via forming a cartilage defect on the femoral trochlea. Figure 4A displays the results of a 4-week treatment in the presence/absence of Fe_3_O_4_-CS/KGN (n = 5). For the PBS group, a significant edema signal was observed via MRI (MRI, Bruker 70/20 UR, Germany), which was abnormally brighter than the surrounding tissue (blue arrow), indicating postoperative complications. Compared with the PBS treated group, the KGN-incubated ADSCs group (KGN group) did not show significant edema signals. However, the defect position was difficult to discern because of the surrounding unaffected tissue. This may be the result of favorable healing, or difficult identification due to limitation of the MR instrument. In the Fe_3_O_4_-CS/KGN group, the defect position was easily identified, and demarcation was clearly observed. The negligible edema signal may be due to KGN improving ADSCs chondrogenesis 23. Its antioxidant properties 39 may also reduce the oxidative stress response caused by trauma or inflammation 40-42, thus protecting ADSCs and chondrocytes from apoptosis. On the other hand, Fe_3_O_4_-CS/KGN played a significant role in brighter T_2_-weighted MR imaging, which was a result of the increased proton transverse relaxation. The MR signals from the defect (red circle) were thus distinct from adjacent tissue and highlighted the defect boundaries. From micro-CT diagnosis (Figure 4B, Xradia 520 Versa, Zeiss, Germany), we found that part of the newly formed bone trabecula was mineralized underneath the cartilage, indicating that Fe_3_O_4_-CS/KGN MNPs did not suppress osteogenesis in the osteochondral defect model. However, the cartilage layer was not yet completely formed after the first 4 weeks.

After 12-weeks of restoration, the MRI results showed that the recovery was almost complete among the control, KGN alone and Fe_3_O_4_-CS/KGN groups. As shown in Figure 4A (12 W), the edema signals mostly disappeared, while the newly formed cartilage layer was irregular and rough. Compared with the control group, the tissue in the KGN-treated group was more organized, and newly formed bone was visible, but the newly-formed cartilage was faintly rough. As for Fe_3_O_4_-CS/KGN, from the MRI, the new cartilage layer appeared to be integrated and lubricated, and new bone trabecula was reconstituted. Additionally, the enhanced T_2_-weighted contrast signals were almost undistinguishable. Notably, the micro-CT images revealed that the subchondral bone was totally calcified and that there was a clear demarcation line between cartilage and bone (Figure 4B, 12 W), indicating that Fe_3_O_4_-CS/KGN did not inhibit the osteochondral reconstruction.

Throughout the 12-week period, we found that Fe_3_O_4_-CS/KGN MNPs acted as positive contrast agents in the early stage* in vivo* and then were eliminated by metabolic process in the last stage. On one hand, it was believed that Fe_3_O_4_ nanoparticles were removed via a cytoprotective mechanism, and endocytosis caused metal nanoparticles to enter the cytoplasm; however, nanoparticles were still trapped into cytoplasmic vesicles and were divided randomly 43, 44. On the other hand, the self-assembled MNPs could be disintegrated by the low pH environment in the lysosome. These may be the reasons that the T_2_-weighted signals were reduced over the 12-week period. The results indicated that the metal nanoparticles were eliminated and that they were verified to be safe and biocompatible.

### Histological assessment

**Figure 5:**
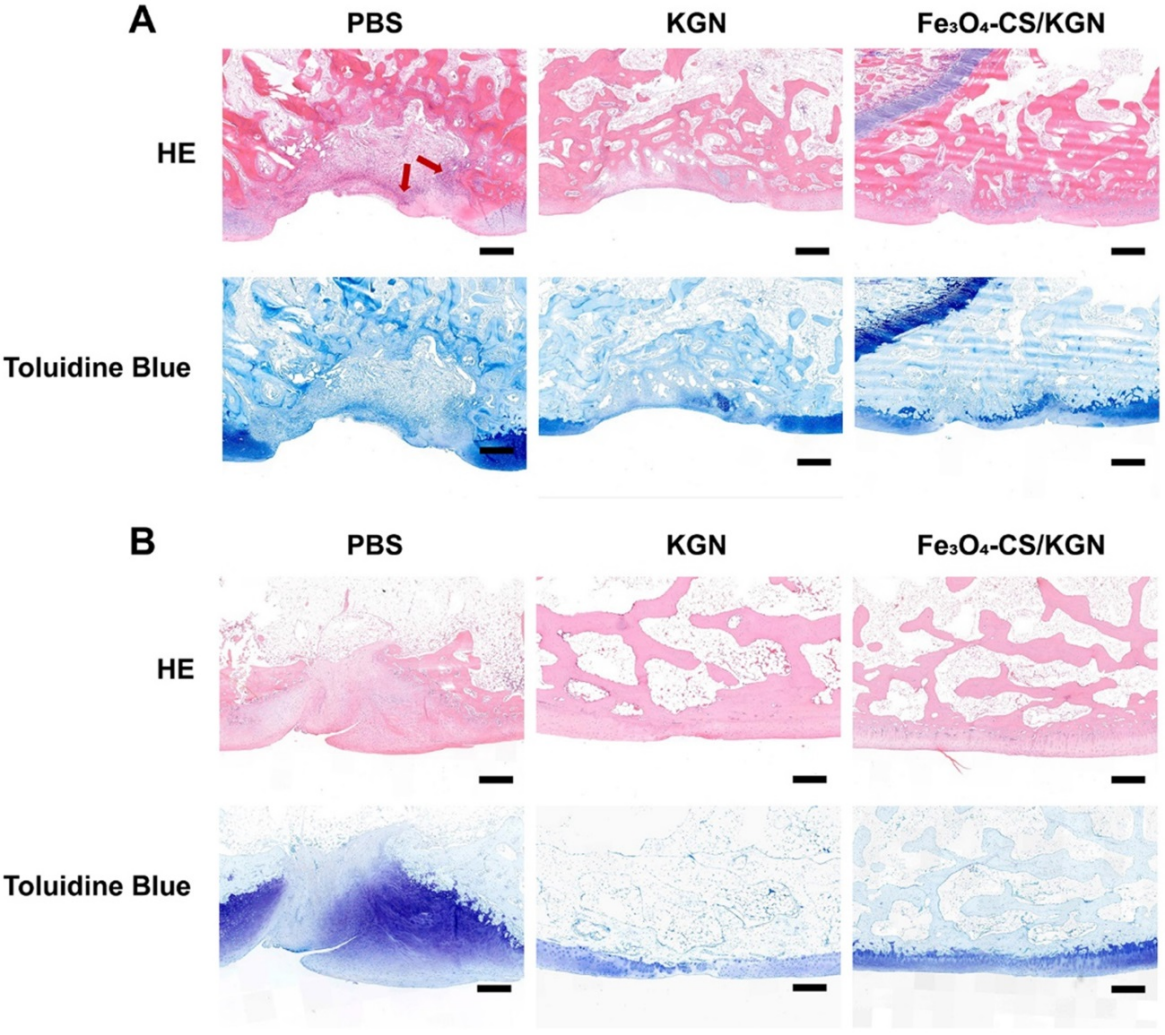
** Histologic assessments. A)** and** B)** correspond to HE and Toluidine Blue staining of rebuilt osteochondral lesions after 4- and 12-week treatment (red arrow: mononuclear cells; all scale bars are 200 µm).

**Figure 6:**
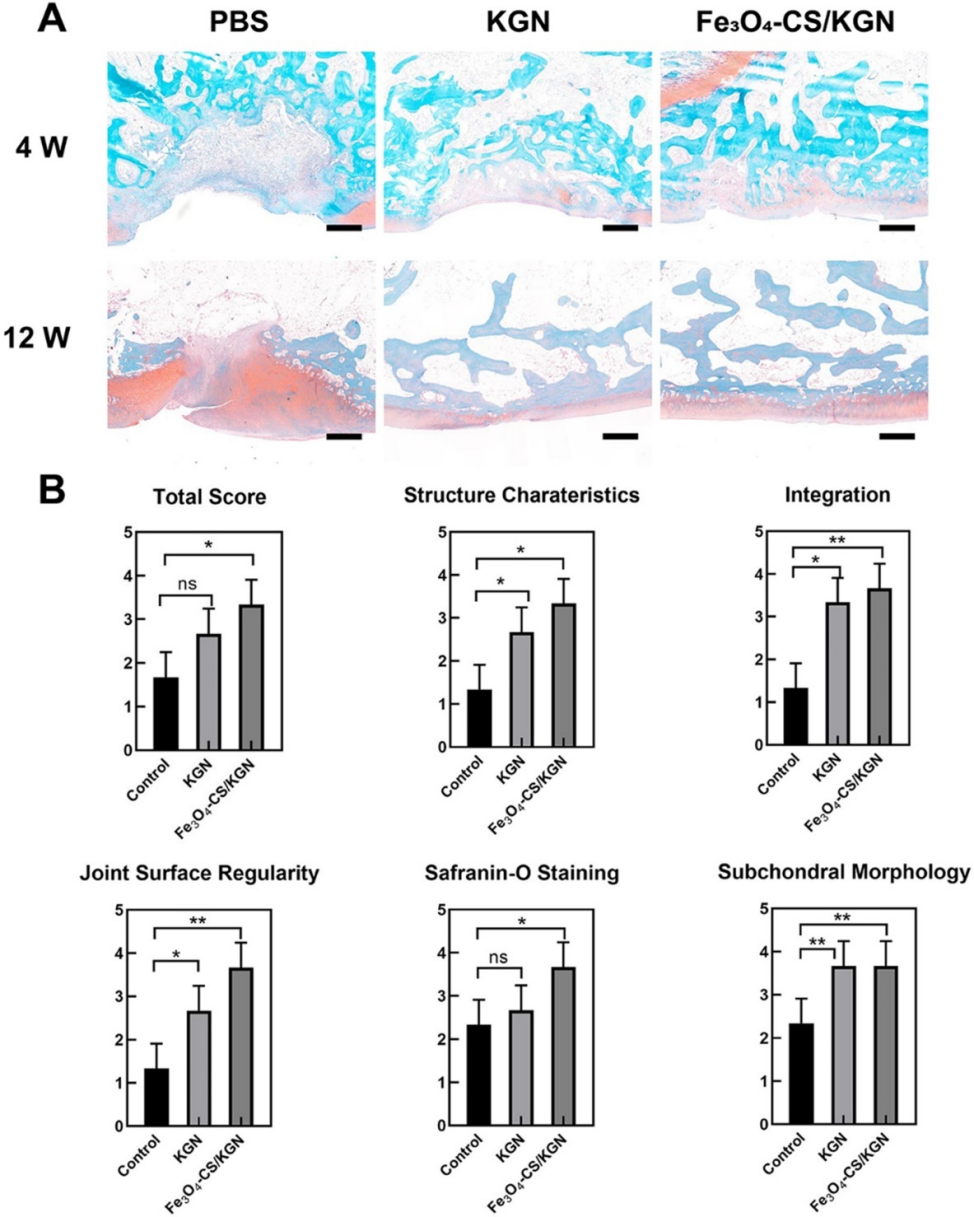
** A)** Safranin-O staining of rebuilt osteochondral lesions (all scale bars are 200 µm); **B)** ICRS scoring (n = 3), * *p* < 0.05, ** *p* < 0.01, ***p < 0.001, ns: not significant.

After 4 weeks, in the PBS group, we observed severe disruption at the site of tissue regrowth accompanied by massive mononuclear cell infiltration (red arrow of Figure 5A, HE staining), and thus severe distinct edema. The nuclei of mononuclear cells were dyed with hematine and were distributed both in the superficial and calcified layers. KGN treatment resulted in the formation of neo-chondrocytes and the appearance of round cells. Extensive mononuclear cell infiltration was not found in the lesion (HE staining), but a new cartilaginous matrix formed at the edge of the defect (toluidine blue staining). However, the boundary between the subchondral bone and the cartilage was still obscure. In comparison, the Fe_3_O_4_-CS/KGN treatment obviously improved the outcome. The HE staining results showed that no small mononuclear cells infiltrated the femoral trochlea, and it was not observed the deterioration of surrounding cartilage due to osteochondral injury. Moreover, the subchondral plate was preliminarily formed together with isogenous chondral groups (toluidine blue). From the pathological assessment, it was possible to determine that the outermost layer was almost completely formed, and homogeneous ECM was encapsulated at the rim of the lesion. Of the above three groups, the lesion demarcation in the Fe_3_O_4_-CS/KGN group was fully integrated. In conclusion, this therapy can effectively promote cartilage formation and osteochondral regeneration. However, Safranin-O staining (Figure 6A) did not yet show normalization due to the lack of sufficient proteoglycans, and additional days were required for restoration.

At 12 weeks (Figure 5B), the three groups showed enhanced healing compared with the 4-week restoration time point. Generally, the PBS group showed a mild sign of repair, such as palingenetic tissue covering the defect, and the massive mononuclear cells disappeared. However, no tidemark or superficial fibrous structure was completely generated, and the unintegrated structure may not transmit the mechanical forces properly. KGN-bearing ADSCs could undergo chondrogenesis in the upper layer, and subchondral ossification took place in the lower cartilage zone, which had a remarkable calcified tidemark. The only drawback was that the superficial chondral layer was thin and unfilled. Nevertheless, the non-lubricated cartilage layer may induce later osteoarthritis (OA) because it cannot transmit stress heterogeneously from various directions 37, 45. In the Fe_3_O_4_-CS/KGN group, an adequately smooth superficial layer was well- integrated with ambient ECM, and the differentiated round cells were orientated with a column arrangement in the underlying lower zone. Safranin-O staining (Figure 6A) showed more homogeneous proteoglycans accumulation, indicating new and intact cartilage formed.

At 12 weeks, International Cartilage Repair Society (ICRS) scores were used for the regenerative evaluation 2. Significant improvements were observed in the Fe_3_O_4_-CS/KGN group (Figure 6B). Histological scoring evaluation was used to assess the osteochondral regeneration from six aspects, including total score, structure characteristics, integration, joint surface regularity, Safranin-O staining and subchondral morphology. As a result, the Fe_3_O_4_-CS/KGN group had generally higher scores than the other groups.

Overall, compared with the 4- and 12-week restoration results, an improved therapeutic effect was obtained, especially the Fe_3_O_4_-CS/KGN MNPs group, which exhibited an intact chondral/ subchondral structure, lubricated superficial layer and demarcated integration. It is worth mentioning that newly differentiated chondrocytes will secrete more ECM if left for several weeks. Thus, the newly formed cartilage will be closer to native tissue. However, it is still unclear how this carrier simultaneously regulates chondrogenesis and osteogenesis.

## Conclusion

In summary, we designed and prepared a nanoprobe consisting of chitosan covalently-coupled Fe_3_O_4_ oleic acid nanoparticles that were induced to self-assemble in the presence of negative charge- bearing KGN (Fe_3_O_4_-CS/KGN MNPs). This material showed its enhanced and bright T_2_-weighted contrast performance in MRI and improved the regeneration of osteochondral defects. Differing from simply KGN-induced chondrogenesis, this study systematically demonstrated that the positive MR contrast agent, Fe_3_O_4_-CS/KGN, not only intensified cellular uptake and dramatically strengthened Col Ⅱ secretion *in vitro*, but also improved *in vivo* MR T_2_-weighted contrast imaging of lesions. It also cooperatively enhanced the potential of KGN for inducing chondroid differentiation without exerting an inhibitory influence on subchondral formation. Furthermore, the ADSCs in combination with biocompatible Fe_3_O_4_-CS/KGN nanoprobes as catabolic materials that can be eliminated by the body. This novel magnetic carrier provides a noninvasive approach for *in vivo* diagnosis and treatment of complex joint cartilage damage.

## Materials and Methods

### Preparation of Fe_3_O_4_-CS/KGN MNPs

To a 100 μL 10 mg/mL Fe_3_O_4_-oleic acid solution (Ocean, USA) was added 5 mL N, N-dimethylformamide (DMF), and the mixed solution was stirred at room temperature for 1 h. Then 10 mg sulfo-HOSu (Aladdin, China) and 10 mg EDC hydrochloride (Aladdin, China) were dispersed in 3 mL DMF, which was added into the above solution. Subsequently, a catalytic amount of triethylamine (TCI, Japan) was employed as an additive to activate the reaction. Over 24 h, the residues were purified by DMF and 0.1% acetic acid (Aladdin, China) under a magnetic field. The purified MNPs were re-suspended in 1% CS (Aladdin, China)/acetic acid solution and allowed to react for another 24 h to form Fe_3_O_4_-CS nanoparticles. The complex MNPs were further purified via a dialysis tube (MW = 300 KDa) for 48 h.

To obtain Fe_3_O_4_-CS/KGN self-assembly, 3 mg KGN was firstly dissolved in 0.5 mL DMSO and then was dropped into Fe_3_O_4_-CS solution, and kept stirring for 24 h. Finally, to remove extra KGN, the mixture was dialyzed against water for 48 h (MW = 3 KDa).

### *In vitro* relaxation time and MRI study

To explore the property of Fe_3_O_4_-CS/KGN MNPs, the relaxation time of MNPs was assessed, and an *in vitro* MR imaging study was performed. A series of concentrations, 0.10 mM, 0.08 mM, 0.06 mM, 0.05 mM and 0 mM were used for measuring the *r_2_* value (n = 3) in a 0.5 T magnetic resonance scanner. The parameters were follows: flip angle = 90^o^, TR = 1800 ms, TE = 18.2 ms, and RG = 26 dB.

### Rabbit ADSC extraction and identification

The animal experiments were approved and in accordance with instructions of the Institute Animal Use and Care Committee of Shanghai Jiao Tong University. Rabbit ADSCs were extracted from the abdominal fat of 2.5-2.7 kg rabbits. Briefly, when the rabbits were anesthetized, the abdomen hair was removed and the skin sterilized. The extracted fat was peeled from the abdomen using sterilized surgical instruments. The fat tissue was cut into small pieces and digested in 0.1% Type 1 Collagenase (Gibco, USA) for 45 min at 37 ℃. The suspension was filtered via cell strainers (100 μm, BD, USA) to obtain filter liquid, which was centrifuged at 2000 rpm for 10 min. After discarding the supernatant, the residues were re-suspended with F-12 medium (Gibco, USA) containing 10% fetal bovine serum (FBS, Gibco, USA) and seeded in culture flasks. The complete medium was replaced every 3 days until 70-80% cell attachment was reached.

In order to characterize the extracted cellular phenotype, FCM was used to identify ADSCs. In this study, BB770-conjugated anti-CD44 (BB770-CD44), PE-conjugated anti-CD90 (PE-CD90) and APC-conjugated anti-CD11b (APC-CD11b, BD, USA) antibodies were used for phenotype identification. The procedure was performed according to the manufacturer's specifications. ADSCs at passage 3-5 were employed in all the *in vitro*/*vivo* experiments.

### Cellular cytotoxicity and apoptosis assay

Firstly, the viability of ADSCs after exposure to Fe_3_O_4_-CS/KGN was assessed. Briefly, ADSCs were seeded in a 96-well plate at a concentration of 3 × 10^4^ cells/mL (100 μL per well, n=5) in F12 medium with 10% FBS. After 12-h culture, the F12 complete medium was replaced with fresh medium containing 10 μM, 1 μM or 100 nM KGN or Fe_3_O_4_-CS/KGN, and PBS as a blank control. The medium was discarded after 24 h and fresh medium with 10 % CCK-8 was added. After incubating for 1-2 h, the absorbance was detected using a microplate reader at a wavelength of 450 nm.

Additionally, for the apoptosis assay, the Annexin-FITC/PI kit (Yeasen, China) was employed to investigate the cytotoxicity of Fe_3_O_4_ MNPs. Rat ADSCs were seeded in 6-well plates at a density of 1 × 10^5^ cells/mL (n=3). When the ADSC attachment reached 60-70%, F12 containing 100 nM, 1 μM or 10 μM Fe_3_O_4_-CS/KGN was added to the wells and the cells were cultured for 4 h (n=3), then the medium was replaced with chondrogenic inducing medium for 24 h. Furthermore, PBS treatment was used for a control group. Following this, ADSCs were dissociated using 0.25% trypsin-EDTA and washed three times with PBS. The procedure was performed according to the manufacturer's instructions. The prepared cell samples were detected via FCM within 30 min.

The experimental procedure for live/dead cell staining with Calcein-AM/PI double staining was performed following the apoptosis assay procedures listed previously, and the staining was performed according to the kit instructions (Dojindo, Japan).

### Cellular internalization

ADSCs were seeded in Ibidi dishes (Germany) at a density of 5 × 10^4^ cells/dish (n= 3). After 12h, 2 mL fresh medium with 20 μg/mL calcein or Fe_3_O_4_-CS/calcein was added to the dishes after discarding the supernatants. The cells were collected at 6 h, 12 h and 24 h, respectively. After washing with PBS 3 times, at room temperature, 4% paraformaldehyde was used to fix the cells for 10 min. Then, 1 μg/mL DAPI was used to stain the cell nuclei (2 mL) for 10 min. The prepared samples were kept in PBS and observed via LSCM 33.

### Chondrogenic and osteogenic differentiations

ADSCs were seeded at a density of 5 × 10^4^ cells/dish in Ibidi dishes (Germany), and cultured overnight. The supernatant was discarded and replaced with chondrogenic or osteogenic stimulating medium (Cyagen, USA) containing 10 μM KGN or Fe_3_O_4_-CS/KGN. After 4 h, both the KGN-containing medium was removed and replaced with the corresponding simulating medium for a further 48 h. After the procedure was continued for 2 weeks (chondrogenic differentiation) 2 or 4 weeks (osteogenic differentiation) 46, the anti-type 2 collagen (Novus, USA) antibody was used for chondrogenesis immunofluorescence, and Alizarin Red-S (Cyagen, USA) was used to stain the newly formed calcium nodules, indicating osteogenic differentiation.

### Animal cartilage defect model

To prepare for use in the animal *in vivo* experiments, ADSCs were first treated with 10 μM Fe_3_O_4_-CS/KGN MNPs or KGN for 2 weeks. Briefly, ADSCs at a density of 3 × 10^5^ cells/well were planted on the coverslips of 6-well plates. After 12 h, 10 μM Fe_3_O_4_-CS/KGN MNPs or KGN was added, the cells were further cultured for 4 h, and then the supernatant was discarded. The above procedure was repeated for 14 days, and the medium was replaced every 3 days.

The animal experiments were conducted according to the instructions of the institute Animal Use and Care Committee of Shanghai Jiao Tong University. Generally, 2.5-2.7 kg male rabbits were anesthetized using Zoletil, sterilized, and then the hair from right knees was removed. A defect (4 mm in diameter and 3 mm in depth, n = 5) was formed on the femoral trochlea by removing cartilage and sub subchondral bone. The prepared ADSCs were shaved off from coverslips via a scraper, and the collected ADSCs (appropriately 1 × 10^6^ cells) were deposited in lesions. Defects with PBS treatment were regarded as a control. Specimens in the *in vivo* animal study were evaluated at 4 and 12 weeks. The MR diagnoses were made using the guidance of professional physicians. The parameters of T_2_-weighted imaging were set as follows: Echo Spacing, 8.0 ms; Repetition Time, 2200 ms; Echo Time, 12 ms; FOV = 35 mm ×35 mm.

### Statistical Analysis

Origin 2018 was utilized in diagraph analysis and particle data analysis. *t*-testing statistical analysis was employed to evaluate the experimental data significance. Differences among groups are denoted as ns for not significant, * for *P* < 0.05, ** for *P* < 0.01, and *** for *P* < 0.001. The samples in each test were at least tested three times.

## Supplementary Material

Supplementary figures.

## Abbreviations

ADSC: adipose tissue-derived stem cell
CBFβ: core-binding factor β subunit
Col Ⅱ: type 2 collagen
CS: chitosan
CT: computed tomography
DMF: N, N- dimethylformamide
ECM: extracellular matrix
EDC: 1-(3-dimethylaminopropyl)-3-ethylcarbodiimide hydrochloride
FBS: fetal bovine serum
FCM: flow cytometry
FDA: Food and Drug Administration
Fe_3_O_4_: ferroferric oxide
FT-IR: Fourier transform infrared
GAG: glycosaminoglycans
ICP-MS: inductively coupled plasma-mass
ICRS: International Cartilage Repair Society
KGN: kartogenin
LSCM: laser scanning confocal microscope
MNP: magnetic nanoparticle
MRI: magnetic resonance imaging
MSC: mesenchymal stem cell
NMR: nuclear magnet resonance
PBS: phosphate buffer solution
SIRT1: silent information regulator type 1
sulfo-HOSu: sulfo-N-hydroxy succinimide
TEM: transmission electron microscopy
