# Supplementary material for: Chitosan modified Fe3O4/KGN self-assembled nanoprobes for osteochondral MR diagnose and regeneration
Source: Theranostics. 2020 Apr 15;10(12):5565–77. doi: 10.7150/thno.43569 (PMC7196312; doi:10.7150/thno.43569)
Supplement: Supplementary file 1 — Supplementary figures. [file thnov10p5565s1.pdf]

Supplementary materials

Chitosan modified Fe3O4/KGN self-assembled nanoprobes for osteochondral MR diagnose and regeneration

Yuping Hong[1], Yaguang Han[3], Jun Wu[3], Xinxin Zhao[4], Jin Cheng[1], Guo Gao[1], Qirong Qian[3], Xiuying Wang[5], Weidong Cai[5], Hala Zreiqat[6], Dagan Feng[5], Jianrong Xu[4✉], Daxiang Cui[1,2✉]

1. Institute of Nano Biomedicine and Engineering, Shanghai Engineering Research Centre for Intelligent Diagnosis and Treatment Instrument, Department of Instrument Science and Engineering, School of Electronic Information and Electrical Engineering, Shanghai Jiao Tong University, 800 Dongchuan RD, Shanghai 200240, PR China
2. Institute of Nano Biomedicine, National Engineering Center for Nanotechnology, 28 Jianchuan East RD, Shanghai 200241, PR China
3. Department of Joint Surgery and Sports Medicine, Changzheng Hospital, Second Military Medical University, 415 Fengyang RD, Shanghai 200003, PR China
4. Department of Radiology, Ren Ji Hospital, School of Medicine, Shanghai Jiao Tong University, 160 Pujian RD, Shanghai 200127, PR China
5. School of Computer Science, Faculty of Engineering, University of Sydney, NSW 2006, Australia
6. Murray Maxwell Biomechanics Laboratory, Kolling Institute, Royal North Shore Hospital, University of Sydney, NSW 2065, Australia

✉ Corresponding authors: Daxiang Cui (dxcui@sju.edu.cn) or Jianrong Xu (xujianr@hotmail.com)

1. The NMR spectrum of Fe_3O_4-CS/KGN

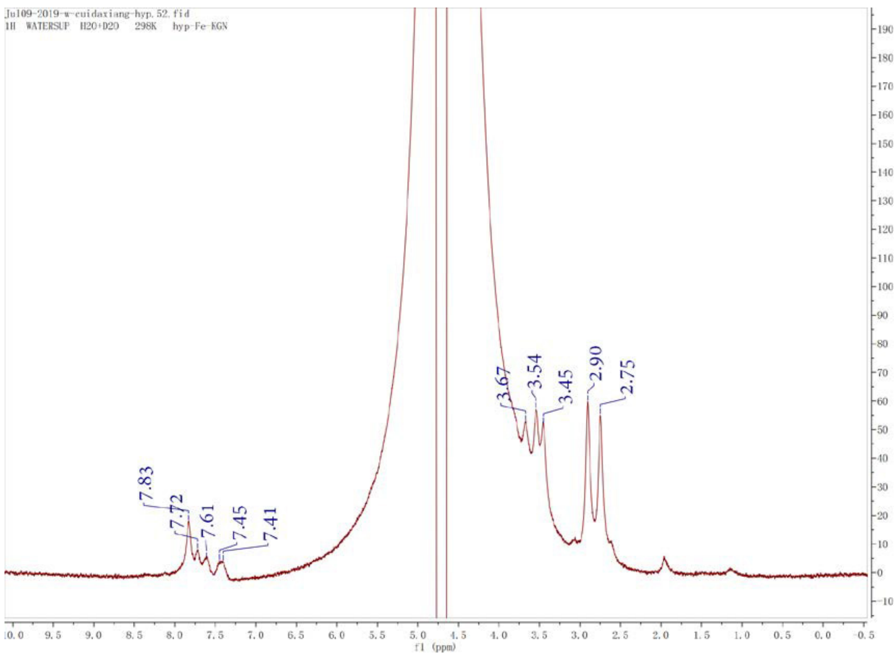

Figure S1. the NMR spectrum of Fe_3O_4-CS/KGN (solvent: H_2O+D_2O, Avance III 600 MHz, Bruker, Germany)

2. Released KGN of Fe_3O_4-CS/KGN

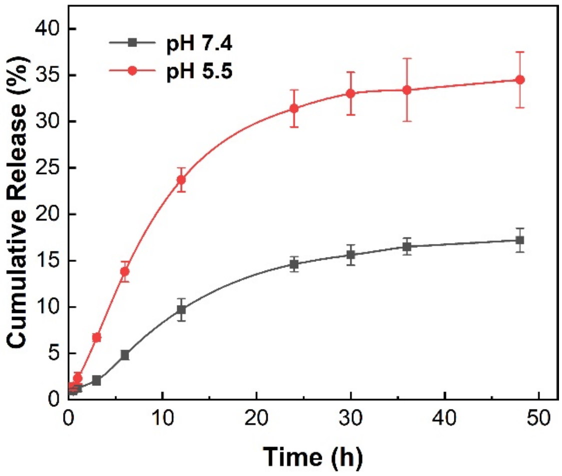

Figure S2. *in vitro* KGN release from Fe_3O_4-CS/KGN nanoparticles in PBS with different pH values (5.5) and pH values (7.4) at 37 °C (n= 3).

3. Characterization of ADSCs

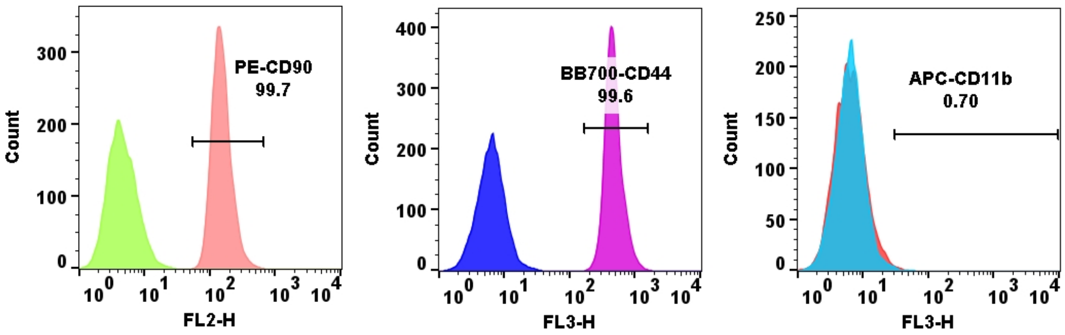

Figure S3. the ADSC characterization of CD90, CD44 and CD11b via FCM

4. The Cell Toxicity

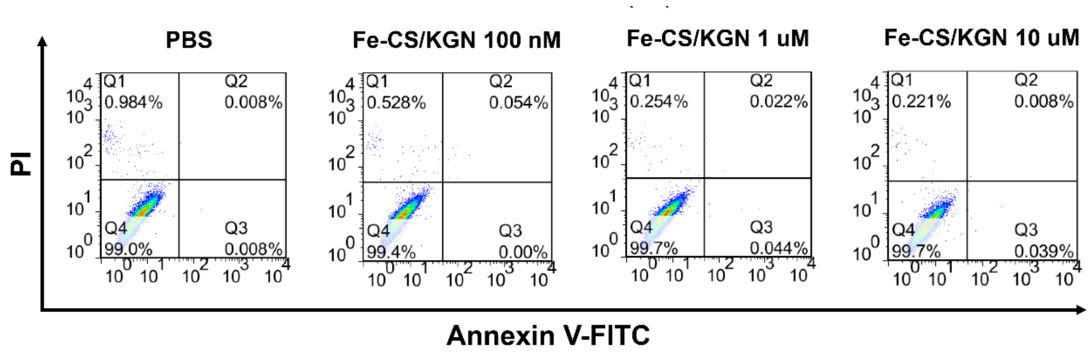

Figure S4. the cell apoptosis after incubated with PBS or different concentrations of Fe_3O_4-CS/KGN via FCM

5. Live/Dead Straining

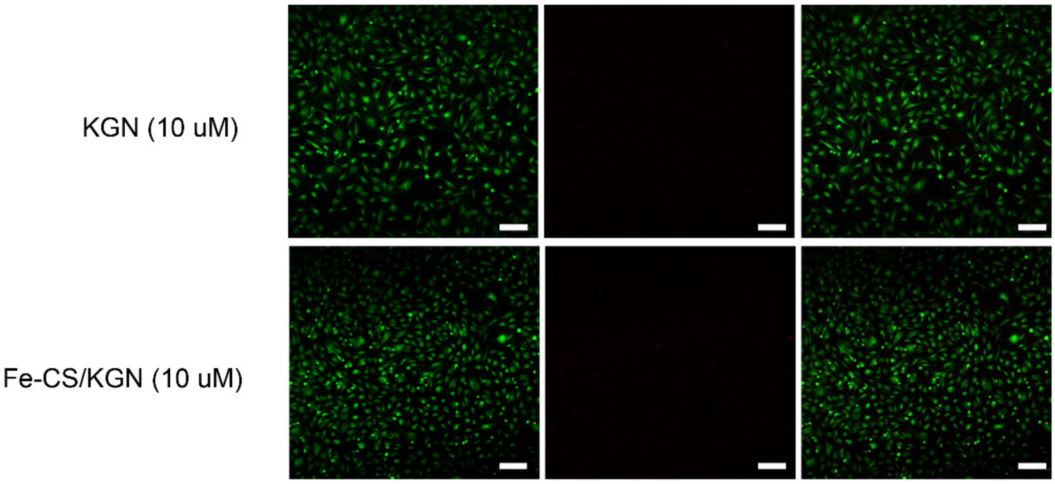

Figure S5. the double staining of Calcein-AM/PI for live/dead cells (scale bar = 100 μm)
